# Supplementary material for: Treatment of Colorectal Cancer in Certified Centers: Results of a Large German Registry Study Focusing on Long-Term Survival
Source: Cancers (Basel). 2023 Sep 15;15(18):4568. doi: 10.3390/cancers15184568 (PMC10526771; doi:10.3390/cancers15184568)
Supplement: Supplementary file 1 [file cancers-15-04568-s001.zip › cancers-2589685-supplementary.pdf]

cancers MDPI

Supplement-Table S1 Full Results of multivariable Cox-regression for all-cause mortality according to diagnosis

		C18 Colon			C20 Rectum		
		HR	Lower 95%-CI	Upper 95%-CI	HR	Lower 95%-CI	Upper 95%-CI
**Treatment in center**	no	1.000			1.000		
	yes	0.921	0.887	0.956	0.978	0.929	1.029
**Sex**	male	1.000			1.000		
	female	0.919	0.885	0.954	0.966	0.916	1.018
**Age at diagnosis**	18- 49	1.000			1.000		
	50-59	1.349	1.198	1.520	1.174	1.016	1.356
	60-69	1.627	1.457	1.818	1.523	1.329	1.746
	70-79	2.496	2.245	2.776	2.505	2.195	2.860
	80+	5.258	4.726	5.851	4.429	3.864	5.076
**Year of diagnosis**	2009-2011	1.000			1.000		
	2012-2014	1.022	0.979	1.066	1.031	0.974	1.091
	2015-2017	1.133	1.074	1.196	0.997	0.923	1.076
**UICC stage**	I	1.000			1.000		
	II	1.230	1.142	1.325	1.540	1.384	1.714
	III	1.456	1.347	1.574	1.431	1.297	1.580
	IV	4.724	4.394	5.078	4.361	3.948	4.816
	X/n.s.	1.996	1.838	2.167	2.178	1.946	2.438
**Grade**	G1	1.000			1.000		
	G2	1.086	0.986	1.197	1.276	1.108	1.470
	G3/4	1.419	1.283	1.570	1.723	1.485	2.000
	GX/n.s.	1.088	0.973	1.218	1.105	0.946	1.290
**Lymphatic invasion**	L0	1.000			1.000		
	L1	1.198	1.138	1.260	1.385	1.284	1.493
	LX/n.s.	1.942	1.717	2.196	2.478	2.097	2.929
**Vein invasion**	V0	1.000			1.000		
	V1/2	1.317	1.247	1.391	1.360	1.248	1.481
	VX/n.s.	1.224	1.089	1.375	1.189	1.010	1.398

n.s.: not spezified

Supplement-Table S2 Sensitivity analyses: Unadjusted and adjusted* hazard ratios with 95%-CI for all-cause mortality following treatment in GCS-certified colorectal cancer centers compared to treatment in non-certified hospitals
* adjusted for age, sex, year of diagnosis, UICC-stage, grade, lymphatic, and vein invasion

	**HR**	**Lower 95%-CI**	**Upper 95%-CI**
**C18 Colon all stages excl. unknown stage univariable**	0.889	0.855	0.924
**C18 Colon all stages excl. unknown stage multivariable**	0.908	0.873	0.945
**C20 Rectum all stages excl. unknown stage univariable**	0.843	0.799	0.890
**C20 Rectum all stages excl. unknown stage multivariable**	0.961	0.909	1.014
